# Regulatory Variation at TERT and TERC Shows Limited Association with Early-Onset Alzheimer’s Disease in Carriers of the Mexican Founder Mutation *PSEN1* A431E

**DOI:** 10.3390/medsci14020228

**Published:** 2026-04-30

**Authors:** Celeste Patricia Gazcón-Rivas, Iliannis Yisel Roa-Bruzón, Luis Félix Duany-Almira, Cesar Aly Valdéz-Gaxiola, Sofia Dumois-Petersen, Luis Eduardo Figuera-Villanueva, Antonio Quintero-Ramos, Carmen Magdalena Gurrola-Díaz, Daniel Ortuño-Sahagun, Yeminia Valle, Oscar Arias-Carrión

**Affiliations:** 1 Programa de Doctorado en Genética Humana, Departamento de Biología Molecular y Genómica, Centro Universitario de Ciencias de la Salud (CUCS), Universidad de Guadalajara, Guadalajara 44340, Jalisco, Mexico; celeste.gazcon1117@alumnos.udg.mx (C.P.G.-R.); iliannis.roa8556@alumnos.udg.mx (I.Y.R.-B.); luis.duany8455@alumnos.udg.mx (L.F.D.-A.); cesar.valdez2320@alumnos.udg.mx (C.A.V.-G.); 2 Instituto de Neurociencias Traslacionales, Departamento de Neurociencias, Centro Universitario de Ciencias de la Salud (CUCS), Universidad de Guadalajara, Guadalajara 44340, Jalisco, Mexico; daniel.ortuno@academicos.udg.mx; 3 Instituto de Investigación en Ciencias Biomédicas, Departamento de Clínicas Médicas, Centro Universitario de Ciencias de la Salud (CUCS), Universidad de Guadalajara, Guadalajara 44340, Jalisco, Mexico; 4 División de Genética, Centro de Investigación Biomédica de Occidente (CIBO), Centro Médico Nacional de Occidente (CMNO), Instituto Mexicano del Seguro Social (IMSS), Guadalajara 44340, Jalisco, Mexico; luis.figuera@academicos.udg.mx; 5 Centro Universitario de los Valles, Departamento de Ciencias de la Salud, Universidad de Guadalajara, Guadalajara 46708, Jalisco, Mexico; sofia.dumois@academicos.udg.mx; 6 Instituto de Investigación en Inmunología, Departamento de Fisiología, Centro Universitario de Ciencias de la Salud (CUCS), Universidad de Guadalajara, Guadalajara 44340, Jalisco, Mexico; antonio.qramos@academicos.udg.mx; 7 Instituto de Investigación en Enfermedades Crónico Degenerativas, Centro Universitario de Ciencias de la Salud (CUCS), Departamento de Biología Molecular y Genómica, Universidad de Guadalajara, Guadalajara 44340, Jalisco, Mexico; carmen.gurrola@academicos.udg.mx; 8 División de Neurociencias Clínica, Instituto Nacional de Rehabilitación Luis Guillermo Ibarra Ibarra, Mexico City 14389, Mexico; 9 Tecnológico de Monterrey, Escuela de Medicina y Ciencias de la Salud, Mexico City 14380, Mexico

**Keywords:** early-onset Alzheimer’s disease, *PSEN1*, telomere biology, TERT, TERC, genetic modifiers

## Abstract

**Background**: Early-onset Alzheimer’s disease (EOAD) caused by autosomal dominant mutations provides a deterministic framework for investigating genetic modifiers of neurodegeneration. Telomere biology has emerged as a central regulator of genomic stability, cellular ageing, and stress response integration, yet its role in EOAD, particularly in under-represented populations, remains poorly defined. **Methods**: We conducted a cross-sectional case–control study to evaluate the genetic distribution, disease association, and predicted regulatory consequences of common variants in the telomere maintenance genes *TERT* and *TERC* in individuals from Western Mexico. The EOAD group comprised genetically confirmed carriers of the *PSEN1* p.Ala431Glu (A431E) founder mutation with clinical EOAD (n = 69), and controls were unrelated individuals without dementia (n = 179). Five common variants were analyzed: rs2242652, rs2853677, rs2736100, and rs10069690 (*TERT*), and rs12696304 (*TERC*). **Results**: Genotype distributions in controls conformed to the Hardy–Weinberg equilibrium. Single-variant analyses showed no significant allele-level associations. Most *TERT* variants did not show significant allele-level associations with EOAD. However, a preliminary genotype-level enrichment for the GC allele at rs12696304 (*TERC*) was observed among EOAD cases compared with controls; allele-level associations were not significant. Linkage disequilibrium analysis revealed low r^2^ values (<0.20), supporting variant independence. Population-level allele frequency comparisons revealed ancestry-dependent divergence across loci; in silico functional annotation localised all variants to non-coding regulatory regions. GTEx-based analyses indicated that rs12696304 acts as an eQTL for *ACTRT3* in whole blood and pituitary, as well as for *LRRC34* in the cerebellar hemisphere, suggesting a potential regulatory network within the TERC locus (3q26.2). **Conclusions**: Overall, common regulatory variants in *TERT* did not show strong independent effects on EOAD susceptibility in *PSEN1 A431E* carriers. However, the convergence of association patterns, functional annotation, and regulatory evidence provides hypothesis-generating support for the *TERC* locus (3q26.2), particularly rs12696304, as a candidate region for further investigation. Additional studies integrating telomere dynamics, functional validation, and multi-omics analyses are needed to clarify the role of telomere biology in the pathogenesis of autosomal dominant EOAD.

## 1. Introduction

Early-onset Alzheimer’s disease (EOAD) is a genetically and biologically distinct form of Alzheimer’s disease (AD), characterised by earlier symptom onset, accelerated progression, and a markedly higher heritable burden compared with late-onset AD (LOAD) [1]. Autosomal dominant EOAD, most commonly driven by pathogenic variants in *PSEN1* (*Presenilin 1*), *PSEN2* (*Presenilin 2*), and *APP* (*Amyloid Beta Precursor Protein*), provides a deterministic model of neurodegeneration that has reshaped mechanistic interpretations of AD, particularly those involving γ-secretase dysfunction and amyloid precursor protein (APP) processing [2,3].

Although these monogenic EOAD mutations have high penetrance, they exhibit substantial clinical heterogeneity, suggesting that secondary molecular or genetic factors modulate disease expression. The *PSEN1* c.1292C>A (p.Ala431Glu) variant (A431E), a founder mutation common in families from Western Mexico, illustrates this paradox. Carriers develop symptoms at an unusually early age and experience rapid disease progression with a notably broad phenotype that frequently extends beyond memory impairment to include motor abnormalities, neuropsychiatric disturbances, and systemic manifestations [4,5]. This multisystem presentation challenges reductionist, neuron-centric frameworks and suggests the involvement of convergent ageing-related cellular processes.

In addition to amyloidogenic mechanisms, *PSEN1* mutations cause widespread cellular dysfunction, including mitochondrial impairment, disrupted calcium homeostasis, oxidative stress, and altered proteostasis [6,7]. These processes overlap with pathways that govern cellular senescence and tissue vulnerability, providing a biologically coherent rationale for investigating modulators of biological ageing in EOAD [8,9,10].

Telomere biology has emerged as a fundamental regulator of genomic stability and cellular longevity. Telomeres progressively shorten with replicative history and cumulative oxidative damage, while telomerase activity—mediated by the catalytic subunit TERT (Telomerase Reverse Transcriptase) and the RNA template TERC (Telomerase RNA Component)—preserves chromosomal integrity [11,12]. Accelerated telomere attrition has been associated with chronic inflammation, mitochondrial dysfunction, and age-related diseases, including neurodegeneration [13].

Genetic variation within *TERT* and *TERC* contributes substantially to interindividual differences in telomere length (TL). Genome-wide association studies (GWAS) have consistently identified common polymorphisms at these loci, predominantly within regulatory non-coding regions [14,15,16]. Although these variants have been extensively investigated in oncological and cardiovascular contexts, their contribution to neurodegenerative phenotypes remains incompletely defined.

Epidemiological studies have reported an association between shorter leukocyte TL and increased dementia risk [8], and Mendelian randomisation analyses suggest that genetically predicted longer TL may confer partial protection against AD [17]. However, these findings derive predominantly from LOAD cohorts of European ancestry, limiting inference for EOAD and ancestrally diverse populations.

This limitation is particularly significant for Latin American populations, whose genomic architectures reflect complex admixture and distinct allele-frequency distributions [18,19]. Mexico is an example of this diversity and harbours one of the most recognisable EOAD founder mutations worldwide. Nevertheless, modifier-focused neurogenetic investigations in this population remain scarce. Whether common TL-associated variants exert disease-modifying effects within deterministic EOAD contexts—especially among *PSEN1* A431E carriers—remains unknown [20].

Furthermore, although TL has been proposed as a biomarker of biological ageing [21], the regulatory landscape through which *TERT* and *TERC* variants may influence EOAD susceptibility has not been systematically evaluated. Disentangling the population distribution, association profiles, and predicted functional consequences of these variants is essential for integrating telomere biology into EOAD pathophysiological frameworks.

In this study, we examine five common variants within *TERT* and *TERC*, assessing their genetic distribution, association with EOAD, and predicted regulatory effects in individuals from Western Mexico, including carriers of the *PSEN1* A431E founder mutation. By investigating ageing-linked genomic mechanisms within a mutation-defined neurodegenerative model, this study expands the ancestry-informed landscape of AD genetics and reframes telomere biology as a potential modifier axis rather than a peripheral correlation.

## 2. Materials and Methods

### 2.1. Study Design

**Figure 1:**
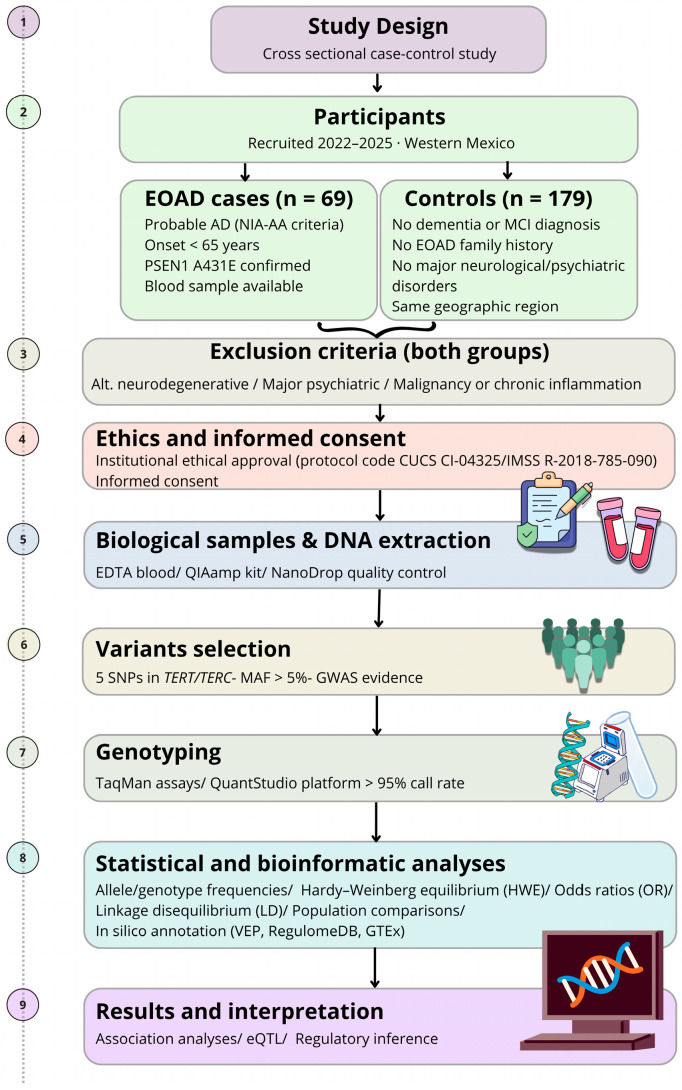
Study design and analytical workflow. Schematic overview of the cross-sectional case–control study conducted in Western Mexico (2022–2025). Participants included individuals with early–onset Alzheimer’s disease (EOAD; *n* = 69) and cognitively healthy controls (*n* = 179) from the same geographic region. EOAD diagnosis was established according to NIA–AA criteria, with PSEN1 A431E mutation status confirmed and blood samples available for all cases. Controls had no dementia, no family history of EOAD, and no major neurological or psychiatric disorders. Shared exclusion criteria included alternative neurodegenerative conditions, major psychiatric illness, malignancy, or chronic inflammatory disease. Following institutional ethical approval and informed consent, peripheral blood samples were collected for DNA extraction (EDTA; QIAamp protocol) with quality control by NanoDrop (Thermo Fisher Scientific, Waltham, MA, USA). Variant selection focused on five SNPs within *TET1/TERC* loci (minor allele frequency >5%) supported by GWAS evidence. Genotyping was performed using TaqMan assays on a QuantStudio platform (>95% call rate). Statistical analyses included allele and genotype frequency estimation, Hardy–Weinberg equilibrium testing, odds ratio calculation, and linkage disequilibrium analysis, complemented by population comparisons. Functional annotation was conducted in silico (VEP, RegulomeDB, GTEx). Downstream analyses included association testing, expression quantitative trait loci (eQTL) integration, and regulatory inference. Created with Canva Pro (Canva Pty Ltd., Sydney, Australia).

This study employed a cross-sectional analytical case–control design to investigate the association between common genetic variants in the telomere maintenance genes *TERT* and *TERC* and EOAD. The analytical approach included comparisons of genotype and allele frequencies, analysis of linkage disequilibrium (LD) structure, population-level allele-frequency contrasts using external reference datasets, and in silico functional annotation to assess the predicted regulatory consequences of the analysed variants. The complete study design and analytical workflow are summarized in Figure 1.

### 2.2. Participants

Participants were recruited between 2022 and 2025 in Western Mexico through specialised neurology clinics and community-based sampling. The EOAD group comprised individuals who met established diagnostic criteria for probable AD under the National Institute on Aging and Alzheimer’s Association framework, with documented symptom onset before age 65 and genetic confirmation of carrier status for the *PSEN1* c.1292C>A (p.Ala431Glu; A431E) variant. Eligibility required the availability of peripheral blood samples suitable for genomic DNA extraction. Individuals were excluded if they had alternative neurodegenerative diagnoses, major psychiatric disorders predating cognitive decline, or systemic medical conditions with known substantial effects on telomere biology, including active malignancy or chronic inflammatory disease.

The control group consisted of unrelated individuals recruited from the same geographic region. Controls had no diagnosis of dementia or mild cognitive impairment, no reported family history of EOAD, no major neurological or psychiatric disorders, and provided peripheral blood samples for DNA analysis. Recruitment strategies were designed to minimise population stratification by ensuring comparable regional ancestries across groups.

The final analytical dataset included 69 EOAD cases and 179 controls. The limited availability of carriers of the *PSEN1* A431E founder mutation determined the sample size. A control-to-case ratio greater than 2:1 was used to enhance statistical efficiency under restricted-case recruitment conditions.

### 2.3. Ethics Approval

All study procedures adhered to the principles outlined in the Declaration of Helsinki (2013) and complied with national ethical and regulatory standards [22]. The protocol was approved by the institutional Ethics, Research, and Biosafety Committees under identifiers CI-04325 of Centro Universitario de Ciencias de la Salud and IMSS R-2018-785-090. Written informed consent was obtained from all participants or their legally authorised representatives through a structured process that included explicit explanations of genetic analyses, confidentiality safeguards, and the voluntary nature of participation. Genetic data were coded using non-identifiable study-specific identifiers, and analyses were conducted exclusively on de-identified datasets.

### 2.4. Biological Samples and DNA Extraction

Peripheral venous blood samples were collected in EDTA-containing tubes and processed within four hours. Genomic DNA was extracted using the QIAamp DNA Blood Mini Kit (QIAGEN GmbH, Hilden, Germany) according to the manufacturer’s standardised protocol. DNA concentration and purity were assessed by spectrophotometry using a NanoDrop instrument (Thermo Fisher Scientific, Waltham, MA, USA), with acceptable purity defined as A260/A280 ratios between 1.8 and 2.0. DNA integrity was verified by agarose gel electrophoresis. Extracted DNA samples were stored at −20 °C until genotyping.

### 2.5. Variant Selection

Variants were selected based on minor allele frequencies greater than 5% in publicly available genomic databases, localisation within intronic or regulatory non-coding regions, prior genome-wide association study (GWAS) evidence linking loci to telomere length or telomerase-related regulation, and the availability of functional annotations in Ensembl, RegulomeDB, and GTEx resources. The analysed variants included rs2242652, rs2853677, rs2736100, and rs10069690 within *TERT*, and rs12696304 within *TERC*.

### 2.6. Genotyping

Genotyping was performed using TaqMan SNP Genotyping Assays (Applied Biosystems, Thermo Fisher Scientific Waltham, MA, USA) with real-time PCR allelic discrimination on a QuantStudio platform (Thermo Fisher Scientific, Waltham, MA, USA). Reactions were conducted in a total volume of 10 µL containing TaqMan Genotyping Master Mix (Thermo Fisher Scientific, Waltham, MA, USA), variant-specific TaqMan assay probes, and 10–20 ng of genomic DNA. Thermal cycling conditions followed the manufacturer’s recommendations: an initial denaturation at 95 °C for 10 min, followed by 40 amplification cycles with denaturation at 95 °C for 15 s and annealing/extension at 60 °C for 1 min [23,24].

Quality control procedures included negative (no-template) controls on each reaction plate, genotype-positive controls where available, and regenotyping of 10% of samples selected at random. Genotype call rates were required to be at least 95%, and concordance rates between duplicate samples exceeded 99%. Genotype clustering outputs were visually inspected to confirm discrimination accuracy. Samples failing quality thresholds were excluded before analysis.

### 2.7. Statistical Analysis

All statistical analyses were conducted using RStudio (version 4.4.1; Core Team, Vienna, Austria, within the RStudio interface 2024.09.1+394 Posit, PBC, Boston, MA, USA). Allele and genotype frequencies were estimated by direct counting. Hardy–Weinberg equilibrium (HWE) was assessed exclusively in the control group using chi-square tests. Differences in allele and genotype distributions between EOAD cases and controls were evaluated using chi-square or Fisher’s exact tests as appropriate.

Genetic associations were estimated as odds ratios (ORs) with 95% confidence intervals (CIs) under additive, dominant, and recessive inheritance models [25,26]. Adjusted *p* values below 0.05 were considered statistically significant.

To control for multiple comparisons, the Bonferroni correction was applied based on the number of variants analysed [27]. Continuous variables were analysed using the Mann–Whitney U test when normality assumptions were not met.

Although 69 EOAD patients were included in the main analysis, complete information for all covariates was not available for all cases and controls. Therefore, adjustment for potential confounders using logistic regression could not be performed reliably. This limitation is acknowledged and discussed below. Covariate availability and missing data. Sociodemographic and clinical data were collected for all participants; however, complete covariate information was not available for all individuals. Age at sampling was available for all 69 EOAD cases and all 179 controls. Sex was recorded for all participants. Ancestry was not formally quantified using principal component analysis or admixture estimation; however, all participants were recruited from the same geographic region (Western Mexico) using strategies designed to minimise population stratification. Among EOAD cases, age at symptom onset was available for all 69 individuals, whereas disease duration was available for the majority but not all cases. Data on education level, comorbidities, and medication use were incompletely recorded across both groups, precluding their inclusion as covariates in regression models. The missing data pattern was predominantly non-systematic and related to the retrospective collection of clinical records rather than to disease severity or genotype. Given these constraints, multivariable logistic regression was not performed, as fitting a model with the available covariates would have resulted in an underpowered and potentially unstable model given the case-to-covariate ratio (69 cases; minimum ~10 events per variable recommended for stable logistic regression).

A post hoc power analysis was performed because the sample size (69 EOAD cases and 179 controls) was determined by the rarity of the *PSEN1 A431E* founder mutation. Power was estimated for allelic associations using a two-sided chi-square framework with α = 0.05. Minor allele frequencies observed in controls served as reference parameters.

Statistical power and minimum detectable odds ratios were calculated in R version 4.4.1 (genpwr package) using functions for case–control power estimation under an allelic model. Power was assessed for predefined effect sizes (OR = 1.5, 2.2, 2.6) and for the observed association for rs12696304 (OR = 3.47). Across the five SNPs, the minimum detectable effect size at 80% power ranged from OR ≈ 2.23 to 2.56, depending on minor allele frequency, while power for more modest effects (OR = 1.5) remained low (19–30%). These results indicate that non-significant findings should be interpreted with caution due to limited power to detect small-to-moderate genetic effects.

### 2.8. Linkage Disequilibrium

Linkage disequilibrium among *TERT* variants was evaluated exclusively in controls using D′ and r^2^ statistics [28]. Variants with consistently low r^2^ values (<0.20) were interpreted as weakly correlated, supporting independent variant-level analyses. Haplotype modelling was not performed due to the limited predictive correlation between loci.

### 2.9. Population-Level Allele-Frequency Comparisons

Allele frequencies observed in the Western Mexico cohort were compared with Phase 3 populations from the 1000 Genomes Project. Pairwise population comparisons were performed using chi-square tests applied to 2 × 2 contingency tables, followed by Bonferroni correction (The 1000 Genomes Project Consortium 2015).

### 2.10. In Silico Functional Annotation

Functional annotation was conducted using the Ensembl Variant Effect Predictor (VEP) release 115 to determine genomic localisation and predicted variant consequences; RegulomeDB version 2.2 to assess regulatory potential; and GTEx (v8) (https://gtexportal.org/home/; accessed 6 March 2026) to evaluate tissue-specific expression quantitative trait loci (eQTL) associations. Analyses examined chromatin-state context, predicted regulatory motif overlap, transcription-factor binding implications, and genotype-dependent expression effects in brain cortex tissue. Variants were categorised based on predicted regulatory relevance [29,30].

### 2.11. Data Handling and Reproducibility

All analyses used de-identified datasets. Analytical scripts, variant definitions, and computational workflows are available upon reasonable request to support independent replication and secondary analyses. 

## 3. Results

### 3.1. Cohort Characteristics

The cohort included 69 individuals diagnosed with EOAD carrying the *PSEN1* c.1292C>A (p.Ala431Glu; A431E) variant and 179 unrelated controls recruited from Western Mexico (Table 1). EOAD cases had a median age at sampling of 50 years (interquartile range (IQR) 45–51), while controls had a median age of 47 years (IQR 41–65). Among EOAD cases, the median age at symptom onset was 42 years (IQR 39–45). The mean disease duration at clinical evaluation was 7.45 years. Sex distribution was similar between cases and controls, with no statistically significant differences.

### 3.2. Clinical Features of PSEN1 A431E Carriers

EOAD cases showed heterogeneous clinical manifestations across cognitive, behavioural, motor, and neuropsychiatric domains (Table 2). The most frequently reported features were gait disturbance (62.32%), irritability (60.87%), insomnia (50.72%), emotional lability (42.03%), dyscalculia (36.23%), and impaired insight (36.23%). Motor findings included spasticity (23.19%), rigidity (23.19%), and paraparesis (17.39%). Neuropsychiatric manifestations included depression (31.88%) and anxiety (33.33%).

### 3.3. Genotype Distributions and Hardy–Weinberg Equilibrium

Genotype distributions in the control group did not deviate from Hardy–Weinberg equilibrium for any analysed variant (Table 3, Table 4, Table 5, Table 6 and Table 7), supporting genotyping quality and population stability.

### 3.4. Single-Variant Association Analyses

Allele and genotype frequencies for all variants are summarised in Table 3, Table 4, Table 5, Table 6 and Table 7. For rs2242652 (*TERT*), allelic and genotypic comparisons showed no statistically significant differences between EOAD cases and controls under additive, dominant, or recessive models (Table 3).

**Table 3:** Genetic distribution of rs2242652 (TERT).

Genetic Model	Genetic Parameter	Controls (n = 179), n (%)	EOAD Cases (n = 69), n (%)	*p* Value	OR (95% CI)
Allelic	G	308 (86.03)	117 (85.47)	—	—
	A	50 (13.97)	21 (14.53)	0.7216	1.11 (0.64–1.92)
Genotypic	GG	135 (75.41)	49 (71.01)	—	—
	GA	38 (21.23)	19 (27.54)	0.3376	1.38 (0.73–2.61)
	AA	6 (3.36)	1 (1.45)	0.4592	0.48 (0.05–3.91)
Dominant	GG	135 (75.41)	49 (71.01)	—	—
	GA+AA	44 (24.59)	20 (28.99)	0.4780	1.25 (0.67–2.33)
Recessive	GG+GA	173 (96.38)	68 (98.55)	—	—
	AA	6 (3.62)	1 (1.45)	0.4310	0.42 (0.05–3.59)

Hardy–Weinberg equilibrium in controls: *p* = 0.1222. Values are n (%) unless otherwise indicated. OR, odds ratio; CI, confidence interval; EOAD, early-onset Alzheimer’s disease. For internal comparisons, the reference (“wild-type”) allele was defined as the most frequent allele in the Western Mexico population. International databases may designate alleles based on ancestral state; comparisons were therefore conducted using observed allele frequencies independent of allele naming conventions. — Reference category. OR: odds ratio; CI: confidence interval.

For rs2853677 (*TERT*), allelic comparisons were not significant. Genotype-level analysis showed that the GG genotype was absent among EOAD cases but present in controls (*p* = 0.0478). Under the recessive model, the GG genotype was associated with OR = 0.05 (95% CI 0.003–0.84) (Table 4).

**Table 4:** Genetic distribution of rs2853677 (TERT).

Genetic Model	Genetic Parameter	Controls (n = 179), n (%)	EOAD Cases (n = 69), n (%)	*p* Value	OR (95% CI)
Allelic	A	243 (67.88)	102 (73.91)	—	—
	G	115 (32.12)	36 (26.09)	0.1913	0.75 (0.48–1.16)
Genotypic	AA	86 (48.04)	33 (47.83)	—	—
	AG	71 (39.66)	36 (52.17)	0.3357	1.32 (0.75–2.33)
	GG	22 (12.30)	0 (0.00)	**0.0478**	0.06 (0.003–0.97)
Dominant	AA	86 (48.04)	33 (47.83)	—	—
	AG+GG	93 (51.96)	36 (52.17)	1.0088	0.98 (0.58–1.76)
Recessive	AA+AG	157 (87.70)	69 (100.00)	—	—
	GG	22 (12.30)	0 (0.00)	**0.0376**	0.05 (0.003–0.84)

Hardy–Weinberg equilibrium in controls: *p* = 0.23. Values are n (%) unless otherwise indicated. OR, odds ratio; CI, confidence interval; EOAD, early-onset Alzheimer’s disease. The absence of the GG genotype among EOAD cases should be interpreted cautiously, given the low absolute number of GG carriers. — Reference category. Bold values indicate statistically significant results (*p* < 0.05). OR: odds ratio; CI: confidence interval.

For rs10069690 (*TERT*), allelic and genotypic comparisons showed no statistically significant differences across inheritance models (Table 5). For rs2736100 (*TERT*), allelic and genotypic comparisons showed no statistically significant differences under additive, dominant, or recessive models (Table 6). For rs12696304 (*TERC*), allelic comparisons were not significant. Genotype-level analysis showed enrichment of the GC genotype among EOAD cases compared with controls (57.97% vs. 28.49%), corresponding to OR = 3.47 (95% CI 1.81–6.63, *p* = 0.0002). The dominant model (GC+GG vs CC) yielded OR = 2.33 (95% CI 1.27–4.26, *p* = 0.0062). These genotype-level associations were nominally significant (Table 7).

**Table 5:** Genetic distribution of rs10069690 (*TERT*).

Genetic Model	Genetic Parameter	Controls (n = 179), n (%)	EOAD Cases (n = 69), n (%)	*p* Value	OR (95% CI)
Allelic	C	278 (77.65)	103 (74.64)	—	—
	T	80 (22.35)	35 (25.36)	0.4760	1.18 (0.75–1.87)
Genotypic	CC	112 (62.57)	38 (55.07)	—	—
	CT	54 (30.17)	27 (39.13)	0.1981	1.47 (0.82–2.66)
	TT	13 (7.26)	4 (5.80)	0.8710	0.91 (0.28–2.95)
Dominant	CC	112 (62.57)	38 (55.07)	—	—
	CT+TT	67 (37.43)	31 (44.93)	0.2799	1.36 (0.78–2.39)
Recessive	CC+CT	131 (73.18)	65 (94.20)	—	—
	TT	7 (26.82)	4 (5.80)	0.6830	0.79 (0.25–2.50)

Hardy–Weinberg equilibrium in controls: *p* = 0.080. Values are n (%) unless otherwise indicated. OR, odds ratio; CI, confidence interval; EOAD, early-onset Alzheimer’s disease. — Reference category. OR: odds ratio; CI: confidence interval.

**Table 6:** Genetic distribution of rs2736100 (*TERT*).

Genetic Model	Genetic Parameter	Controls (n = 179), n (%)	EOAD Cases (n = 69), n (%)	*p* Value	OR (95% CI)
Allelic	A	212 (59.22)	78 (56.52)	—	—
	C	146 (40.78)	60 (43.48)	0.5851	1.12 (0.75–1.66)
Genotypic	AA	66 (36.87)	20 (28.99)	—	—
	AC	80 (44.69)	38 (55.07)	0.1633	1.57 (0.83–2.95)
	CC	33 (18.44)	11 (15.94)	0.8253	1.10 (0.47–2.56)
Dominant	AA	66 (36.87)	20 (28.99)	—	—
	AC+CC	113 (63.13)	49 (71.01)	0.2435	1.43 (0.78–2.61)
Recessive	AA+AC	146 (81.56)	58 (84.06)	—	—
	CC	33 (18.44)	11 (15.94)	0.6975	0.86 (0.39–1.86)

Hardy–Weinberg equilibrium in controls: *p* = 0.318. Values are n (%) unless otherwise indicated. OR, odds ratio; CI, confidence interval; EOAD, early-onset Alzheimer’s disease. — Reference category. OR: odds ratio; CI: confidence interval.

**Table 7:** Genetic distribution of rs12696304 (TERC).

Genetic Model	Genetic Parameter	Controls (n = 179), n (%)	EOAD Cases (n = 69), n (%)	*p* Value	OR (95% CI)
Allelic	C	219 (61.17)	78 (56.52)	—	—
	G	139 (38.83)	60 (43.48)	0.3439	1.21 (0.81–1.80)
Genotypic	CC	84 (46.93)	19 (27.54)	—	—
	GC	51 (28.49)	40 (57.97)	**0.0002**	**3.47 (1.81–6.63)**
	GG	44 (24.58)	10 (14.49)	0.9912	1.00 (0.43–2.35)
Dominant	CC	84 (46.93)	19 (27.54)	—	—
	GC+GG	95 (53.07)	50 (72.46)	**0.0062**	**2.33 (1.27–4.26)**
Recessive	CC+GC	135 (75.42)	59 (85.50)	—	—
	GG	44 (24.58)	10 (14.49)	0.0882	0.52 (0.25–1.10)

Hardy–Weinberg equilibrium in controls: *p* = 0.318. Values are n (%) unless otherwise indicated. OR, odds ratio; CI, confidence interval; EOAD, early-onset Alzheimer’s disease. — Reference category. Bold values indicate statistically significant results (*p* < 0.05). OR: odds ratio; CI: confidence interval.

A post hoc power analysis was performed using a two-sided allelic chi-square test framework (α = 0.05) to quantify detectable effect sizes given the available sample (69 EOAD cases and 179 controls). Using the observed MAF in controls as a reference, the study had adequate power (≥80%) to detect relatively large genetic effects, with minimum detectable odds ratios at 80% power ranging from OR = 2.23 to OR = 2.56, depending on SNP-specific MAF. In contrast, power to detect modest effects was low across all variants (19–30% for OR = 1.5), indicating a high probability of type II error for small associations; therefore, non-significant findings should not be interpreted as evidence of absence.

Of the five TERT/TERC variants analyzed, rs12696304 was the only variant showing significant association with EOAD, under both genotypic and dominant models (OR = 3.47, 95% CI 1.81–6.63, and OR = 2.33, 95% CI 1.27–4.26, respectively). Post hoc power for the observed GC genotype effect (OR = 3.47) exceeded 90%, supporting the robustness of this specific association. The absence of significant associations between any of the five variants and the clinical risk factors evaluated within the case group (including diabetes, hypertension, and obesity) further indicates that the identified association is not driven by clinical comorbidities and that these variables do not act as confounders in the present analysis.

To assess potential imbalances in the distribution of cases and controls across demographic strata, a Mantel–Haenszel stratified analysis was performed, adjusting for sex and age, with age dichotomized at 50 years according to the early-onset disease definition. As rs12696304 is an autosomal variant, its genotype is not determined by sex or age; thus, the stratified adjustment addresses potential distributional imbalances between groups rather than suggesting a biological modification of the variant itself. The association remained significant after adjustment for sex (OR = 2.53, 95% CI 1.13–5.67, *p* = 0.021), age (OR = 6.56, 95% CI 1.45–29.72, *p* = 0.007), and combined sex and age adjustment (OR = 5.42, 95% CI 1.23–23.82, *p* = 0.013). The modest change in OR after sex adjustment reflects the nearly balanced sex distribution between groups (43.48% vs. 50.28% male). The more substantial change after age adjustment reflects the distributional asymmetry between cases and controls rather than classical confounding: controls had a wide age range (median 47 years, IQR 41–65), while cases clustered near the dichotomization threshold (median 50 years, IQR 45–51), amplifying stratum-specific OR estimates and contributing to the wide confidence intervals observed in adjusted models. Overall, these findings indicate that the association of rs12696304 with EOAD is not explained by the demographic or clinical variables examined, and the stratified analysis supports rather than undermines the validity of this result. However, given the limited stratum-specific sample sizes, these findings should be considered preliminary and replication in larger cohorts is needed to obtain more stable estimates.

### 3.5. Linkage Disequilibrium Structure

Pairwise linkage disequilibrium analysis among *TERT* variants in controls revealed low r^2^ values (<0.20) across all SNP pairs (see Supplementary Material), indicating weak predictive correlation between loci. D′ values were moderate for selected pairs but did not indicate a strong correlation. Therefore, variants were analysed independently, and haplotype modelling was not performed.

### 3.6. Population-Level Allele-Frequency Comparisons

Allele frequencies in the Western Mexico cohort were compared with Phase 3 populations from the 1000 Genomes Project (Table 8, Table 9, Table 10, Table 11 and Table 12). For rs2242652 (*TERT*), significant differences after Bonferroni correction were observed in Asian, East Asian, and Other Asian populations, while African, European, and Latin American populations did not differ significantly (Table 8).

For rs2853677 (*TERT*), significant differences after correction were detected in African Others, European, Other Asian, and South Asian populations (Table 9). For rs10069690 (*TERT*), significant differences after correction were observed in African, African American, African Others, and Latin American 1 populations (Table 10). For rs2736100 (*TERT*), significant differences after correction were detected in European, South Asian, and Global total comparisons (Table 11). For rs12696304 (*TERC*), significant allele-frequency differences after correction were observed in multiple populations, including African, African American, Asian, East Asian, European, and Latin American 2 groups (Table 12).

**Table 8:** Population-level allele frequency comparison for rs2242652 (*TERT*).

Population	Allele G, n (%)	Allele A, n (%)	*p* Value (Raw)	*p* Value (Bonferroni)
Western Mexico	308 (86.03)	50 (13.97)	—	—
African	17,789 (86.77)	2713 (13.23)	0.743	1.000
African American	17,221 (86.70)	2641 (13.30)	0.771	1.000
African Others	568 (88.75)	72 (11.25)	0.248	1.000
Asian	5365 (78.03)	1513 (21.97)	4.09 × 10^−4^	**0.00491**
East Asian	4233 (77.98)	1195 (22.02)	4.27 × 10^−4^	**0.00512**
European	204,411 (80.58)	49,253 (19.42)	0.0111	0.134
Latin American 1	3580 (81.72)	802 (18.28)	0.0474	0.569
Latin American 2	5171 (86.21)	827 (13.79)	0.987	1.000
Other Asian	1132 (78.07)	318 (21.93)	0.00104	**0.0125**
South Asian	2395 (82.30)	515 (17.70)	0.0915	1.000
Global total	246,997 (81.11)	57,507 (18.89)	0.0209	0.251

Allele frequencies were compared with those from Western Mexico using χ^2^ tests (2 × 2). Bonferroni correction was applied for multiple comparisons. Values are n (%). — Reference population (Western Mexico cohort). Bold values indicate statistically significant differences after Bonferroni correction (*p* < 0.05). Raw *p* values are shown for comparison.

**Table 9:** Population-level allele frequency comparison for rs2853677 (*TERT*).

Population	Allele G, n (%)	Allele A, n (%)	*p* Value (Raw)	*p* Value (Bonferroni)
Western Mexico	115 (32.12)	243 (67.88)	—	—
African	13,737 (27.40)	36,397 (72.60)	0.0529	0.529
African American	13,304 (27.53)	35,022 (72.47)	0.0603	0.603
African Others	433 (23.95)	1375 (76.05)	0.00145	**0.0145**
Asian	3934 (36.72)	6780 (63.28)	0.0854	0.854
East Asian	2946 (35.12)	5442 (64.88)	0.268	1.000
European	184,163 (42.97)	244,457 (57.03)	4.32 × 10^−5^	**4.32 × 10** **^−^** **^4^**
Latin American 1	3373 (36.95)	5755 (63.05)	0.0714	0.714
Latin American 2	5278 (29.90)	12,374 (70.10)	0.395	1.000
Other Asian	988 (42.48)	1338 (57.52)	2.63 × 10^−4^	**0.00263**
South Asian	2093 (57.44)	1551 (42.56)	6.58 × 10^−20^	**6.58 × 10^−19^**

Allele frequencies were compared with those from Western Mexico using χ^2^ tests (2 × 2). Bonferroni correction was applied for multiple comparisons. Values are n (%). — Reference population (Western Mexico cohort). Bold values indicate statistically significant differences after Bonferroni correction (*p* < 0.05). Raw *p* values are shown for comparison.

**Table 10:** Population-level allele frequency comparison for rs10069690 (*TERC*).

Population	Allele C, n (%)	Allele T, n (%)	*p* Value (Raw)	*p* Value (Bonferroni)
Western Mexico	278 (77.65)	80 (22.35)	—	—
African	21,996 (41.85)	30,560 (58.15)	3.0 × 10^−42^	**3.3 × 10** **^−^** **^41^**
African American	21,343 (42.12)	29,329 (57.88)	1.5 × 10^−41^	**1.6 × 10** **^−^** **^40^**
African Others	653 (34.66)	1231 (65.34)	2.4 × 10^−51^	**2.6 × 10** **^−^** **^50^**
Asian	8232 (78.28)	2284 (21.72)	0.828	1.000
East Asian	6512 (78.84)	1748 (21.16)	0.638	1.000
European	339,545 (74.22)	117,967 (25.78)	0.154	1.000
Latin American 1	6000 (66.02)	3088 (33.98)	6.3 × 10^−6^	**6.9 × 10** **^−^** **^5^**
Latin American 2	14,228 (80.90)	3360 (19.10)	0.140	1.000
Other Asian	1720 (76.24)	536 (23.76)	0.604	1.000
South Asian	2701 (75.11)	895 (24.89)	0.317	1.000
Global total	406,539 (71.31)	163,571 (28.69)	0.00946	0.104

Allele frequencies were compared with those from Western Mexico using χ^2^ tests (2 × 2). Bonferroni correction was applied for multiple comparisons. Values are n (%). — Reference population (Western Mexico cohort). Bold values indicate statistically significant differences after Bonferroni correction (*p* < 0.05). Raw *p* values are shown for comparison.

**Table 11:** Population-level allele frequency comparison for rs2736100 (*TERT*).

Population	Allele C, n (%)	Allele A, n (%)	*p* Value (Raw)	*p* Value (Bonferroni)
Western Mexico	146 (40.78)	212 (59.22)	—	—
African	24,699 (46.10)	28,873 (53.90)	0.0499	0.549
African American	23,855 (46.13)	27,853 (53.87)	0.0487	0.536
African Others	844 (45.28)	1020 (54.72)	0.131	1.000
Asian	4739 (42.82)	6327 (57.18)	0.475	1.000
East Asian	3535 (41.31)	5023 (58.69)	0.887	1.000
European	272,889 (50.65)	265,903 (49.35)	2.33 × 10^−4^	**0.00257**
Latin American 1	4605 (48.02)	4985 (51.98)	0.00835	0.0919
Latin American 2	6721 (38.34)	10,811 (61.66)	0.375	1.000
Other Asian	1204 (48.01)	1304 (51.99)	0.0122	0.135
South Asian	5210 (60.64)	3382 (39.36)	9.06 × 10^−14^	**9.97 × 10** **^−^** **^13^**
Global total	329,529 (49.88)	331,067 (50.12)	6.98 × 10^−4^	**0.00768**

Allele frequencies were compared with those from Western Mexico using χ^2^ tests (2 × 2). Bonferroni correction was applied for multiple comparisons. Values are n (%). — Reference population (Western Mexico cohort). Bold values indicate statistically significant differences after Bonferroni correction (*p* < 0.05). Raw *p* values are shown for comparison.

**Table 12:** Population-level allele frequency comparison for rs12696304 (*TERC*).

Population	Allele C, n (%)	Allele G, n (%)	*p* Value (Raw)	*p* Value (Bonferroni)
Western Mexico	219 (61.17)	139 (38.83)	—	—
African	1349 (45.79)	1597 (54.21)	5.1 × 10^−8^	**6.1 × 10^−7^**
African American	1295 (45.73)	1537 (54.27)	4.8 × 10^−8^	**5.8 × 10^−7^**
African Others	54 (47.37)	60 (52.63)	0.0128	0.154
Asian	46 (41.07)	66 (58.93)	2.8 × 10^−4^	**0.0034**
European	11,252 (73.06)	4148 (26.94)	7.9 × 10^−7^	**9.5 × 10^−6^**
East Asian	36 (41.86)	50 (58.14)	0.00174	**0.0209**
Latin American 1	91 (62.33)	55 (37.67)	0.888	1.000
Latin American 2	307 (50.33)	303 (49.67)	0.00136	**0.0163**
Other Asian	10 (38.46)	16 (61.54)	0.038	0.456
South Asian	62 (63.27)	36 (36.73)	0.795	1.000
Global total	13,548 (67.63)	6484 (32.37)	0.0114	0.137

Allele frequencies were compared with those from Western Mexico using χ^2^ tests (2 × 2). Bonferroni correction was applied for multiple comparisons. Values are n (%). — Reference population (Western Mexico cohort). Bold values indicate statistically significant differences after Bonferroni correction (*p* < 0.05). Raw *p* values are shown for comparison.

### 3.7. Functional Annotation and Regulatory Inference

In silico functional annotation localized all analyzed variants to non-coding regions of the genome. RegulomeDB classification predicted regulatory potential for rs2853677 and rs2736100 (*TERT*), while rs12696304 (*TERC*) received a high regulatory probability score (Rank 1a) in RegulomeDB, consistent with (but not confirming) functional regulatory activity.

### 3.8. Expression Analyses

**Figure 2:**
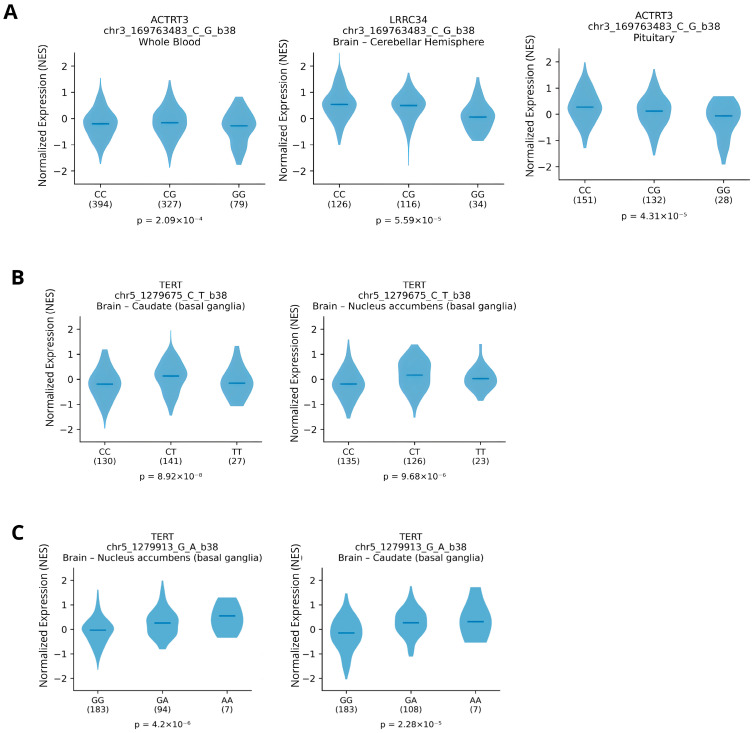
eQTL analysis of *TERT* and *TERC* variants. (**A**) rs12696304 (TERC) showing a dose-dependent effect on ACTRT3 and LRRC34 expression in whole blood, pituitary, and cerebellar hemisphere. (**B**) rs10069690 (TERT) is associated with significant differences in TERT expression in the caudate and nucleus accumbens. (**C**) rs2242652 (TERT) showing transcriptional modulation of TERT in the basal ganglia. *p*-values indicate statistically significant associations between genotype and gene expression levels. This figure was generated using RStudio (version 2024.09.1+394).

GTEx-based analyses revealed genotype-dependent differences in expression across multiple tissues (Figure 2). The variant rs12696304 (*TERC*) was identified as a significant eQTL for *Actin Related Protein T3* (*ACTRT3*) in whole blood and pituitary and for Leucine Rich Repeat Containing 34 (*LRRC34*) in the cerebellar hemisphere. This variant showed a consistent allele-dose pattern (CC > CG > GG), with the G allele associated with reduced gene expression (*p* ≈ 10^−4^–10^−5^). These expression associations are derived from population-level reference data in GTEx and may not reflect the regulatory landscape in *PSEN1* A431E-associated EOAD tissue. In addition, these eQTL associations are derived from population-level reference datasets and have not been experimentally validated in disease-relevant neural contexts, particularly in tissues affected by *PSEN1 A431E*–associated EOAD. Therefore, while these data provide supportive regulatory annotations, they cannot be assumed to represent gene–expression dynamics in the specific pathological environment examined in this study.

In addition, the *TERT* variants rs10069690 and rs2242652 exhibited significant eQTL effects in basal ganglia regions, including the caudate and nucleus accumbens. For rs10069690, a genotype-dependent pattern (CC, CT, TT) was observed with significant differences in TERT expression (*p* ≈ 10^−6^–10^−8^). Similarly, rs2242652 showed increased TERT expression in the GA genotype compared with the GG genotype, while the AA genotype—despite the smaller sample size—followed the same trend, with highly significant associations (*p* ≈ 10^−6^–10^−5^).

### 3.9. Predicted Motif Analyses

Sequence motif modelling indicated that the analysed variants overlapped with positions within predicted regulatory motifs to varying degrees (see Supplementary Material). Variants mapping to high-information-content positions showed greater predicted regulatory impact, whereas those at lower-information-content positions suggested more modest functional consequences.

## 4. Discussion

This study investigated whether common regulatory variants in the telomere maintenance genes *TERT* and *TERC* influence disease susceptibility or phenotypic modulation in a genetically defined cohort of EOAD associated with the *PSEN1* p.Ala431Glu (A431E) founder mutation. Several observations emerge.

### 4.1. Demographic Profile and Clinical Characteristics of the Cohort

The demographic data indicate that the age at symptom onset in patients with EOAD was 42 years (IQR 39–45), consistent with reports for pathogenic *PSEN1* mutations, which typically manifest between 30 and 50 years of age and are characterised by aggressive clinical phenotypes and rapid progression [31]. The higher median age at sampling in patients compared to controls likely reflects the interval between symptom onset and clinical diagnosis. Sex distribution was similar in both groups, consistent with previous studies showing that EOAD, unlike LOAD, does not exhibit a marked sex predominance [4,5].

The clinical phenotype associated with *PSEN1* p.Ala431Glu (A431E) in this cohort was broad and multisystemic, extending beyond isolated memory impairment. In addition to cognitive and behavioural symptoms, motor manifestations, such as gait disturbances and pyramidal signs, were frequently observed, consistent with previous descriptions of this mutation [4,5]. These findings support the view that *PSEN1* A431E involves early motor pathway compromise, distinguishing it from sporadic LOAD.

The advanced clinical presentation at evaluation may reflect prolonged exposure to cellular stressors, including oxidative stress and mitochondrial dysfunction previously described in *PSEN1*-related EOAD [6]. These mechanisms are also associated with telomere shortening, providing a biological rationale for examining *TERT* and *TERC* variants as potential modifiers of cellular ageing processes underlying the disease [11].

### 4.2. Association Analysis of TERT and TERC Variants

The joint analysis of common variants in *TERT* and *TERC* suggests that, although none are classified as pathogenic, they may modulate telomere biology in EOAD. Most *TERT* variants evaluated did not show independent associations with EOAD in the bivariate analyses. However, some genotype-specific patterns were observed.

For rs2853677, the absence of the G/G genotype among patients and its exclusive presence in controls suggests a possible protective effect of homozygosity for the G allele. Given the small number of carriers, this observation should be interpreted with caution and considered exploratory. Variants rs2242652, rs2736100 and rs10069690 did not show significant associations in this cohort. Nevertheless, functional evidence reported in the literature and the in silico analyses performed here suggest that these variants may influence telomerase regulation and telomere maintenance [20].

### 4.3. Functional Interpretation of TERT and TERC Variants

Functional evidence from previous studies and bioinformatic analyses indicates that several evaluated variants may affect telomerase regulation. The rs2242652 A allele, located in a regulatory region of *TERT*, has been associated with reduced TERT expression via the generation of truncated transcripts, shorter telomere length, and increased cancer risk [32,33]. The T allele of rs10069690 has been reported in other populations to generate truncated transcripts through alternative splicing, producing a dominant-negative effect on telomerase activity and shorter telomeres [34,35]. Whether this mechanism operates in the present cohort cannot be confirmed in the absence of direct TL measurements.

Other studies have described intronic variants, such as rs2853677 and rs2736100, have been associated with telomere length variation and the risk of degenerative diseases and cancer, possibly through regulatory effects on TERT expression or through linkage disequilibrium with functional variants not yet characterised [32,36,37]. In this study, in silico analysis predicts that these variants localize to chromatin regions with regulatory signatures, including enhancer-like states and chromatin accessibility in multiple tissues, including the frontal cortex.

For *TERC*, the G allele of rs12696304 has been consistently associated with shorter telomeres and reduced telomere maintenance capacity, particularly in hematopoietic cells [35,38].

Overall, in this study, the in silico prediction indicates that the studied variants are primarily located in regulatory regions; however further experimental validation is required in order to confirm functional relevance. Although telomere length was not directly measured in this study, previous evidence suggests that *TERT* and *TERC* variants associated with shorter telomeres may increase the risk of AD and dementia [8,17]. Together, these findings support the hypothesis that these variants may act as modulators of telomerase activity and cellular ageing rather than direct causal determinants of EOAD [39].

### 4.4. rs12696304 in TERC: Association and Functional Implications

Unlike the *TERT* variants, rs12696304 showed a preliminary association with EOAD. The G/C genotype was significantly associated with increased disease risk (OR = 3.47, 95% CI [1.81–6.63], *p* = 0.0002). This variant is located approximately 1.5 kb downstream of *TERC* at the 3q26.2 locus and has been robustly associated with shorter leukocyte telomere length in genome-wide association studies [15,38]. Each copy of the G allele has been previously associated with an estimated reduction of about 75 base pairs in mean telomere length, corresponding to approximately 3.6 additional years of telomere attrition [38]. It should be noted, however, that TL was not directly measured in the present cohort; therefore, whether rs12696304 exerts a comparable effect on TL in PSEN1 A431E carriers from Western Mexico remains to be established.

Although non-coding, our in silico analyses indicate that rs12696304 is located within a highly active regulatory region characterised by multiple epigenomic signals and transcription factor binding sites, including (CCCTC-Binding Factor) CTCF and (RNA Polymerase II Subunit A) POLR2A, suggesting potential involvement in chromatin architecture and transcriptional regulation at the TERC locus 3q26.2. The relevance of this locus extends beyond AD. A longevity genetics study in a Croatian cohort identified rs12696304 as a candidate variant associated with longevity and suggested a potential regulatory impact on *ACTRT3* [40]. Although no significant association with extreme survival was observed in that study, the convergence of evidence from telomere genetics, longevity studies, and the present supports the TERC locus (3q26.2) as a region warranting further investigation.

A formal post hoc power analysis confirmed that, given the available sample size, the study had at least 80% power only to detect relatively large allelic effects (OR ≥ 2.2), and was substantially underpowered for modest associations (OR ≈ 1.5; power 19–30%). Therefore, the findings should be interpreted as hypothesis-generating. The statistically significant genotype-level association observed for rs12696304 (TERC) corresponds to a large effect size (OR = 3.47), for which post hoc power was high, providing greater confidence in this specific result compared to the null findings for TERT variants. Detecting modest effects typical of complex traits would require much larger sample sizes; for example, achieving 80% power to detect an OR of 1.5 for the least-powered variant (rs2242652) would require approximately 453 cases and 1175 controls, underscoring the need for multi-centre collaborative studies in this genetically rare population.

The genotype-specific association for rs12696304, without a corresponding allelic signal, requires cautious interpretation. This pattern is well described in statistical genetics [25,41] and may indicate a true heterozygote-specific effect or dominance deviation [42], although the possibility of a statistical artifact due to small cell counts cannot be excluded. Independent replication is essential to assess the robustness and biological relevance of this preliminary association.

### 4.5. eQTL Evidence and a Possible Regulatory Network

In our study, eQTL data from GTEx indicate that rs12696304 may influence the expression of additional genes in the 3q26.2 region, including *ACTRT3* and *LRRC34*. Specifically, rs12696304 shows a gene-dosage effect on ACTRT3 expression, with the G allele associated with reduced expression in peripheral blood and pituitary tissue.

*ACTRT3* is a member of the actin-related protein (ARP) family involved in chromatin remodelling complexes such as Switch/Sucrose Non-Fermentable (SWI/SNF), a chromatin remodeling complex which participate in transcriptional regulation and genome stability [43,44]. The observed eQTL signal suggests that rs12696304 may modulate ACTRT3 expression in peripheral blood and pituitary tissue; whether this effect extends to neuronal or EOAD-relevant tissues has not been established. This possibility aligns with evidence suggesting that changes in chromatin accessibility precede transcriptomic alterations in the early stages of AD [45]. However, a direct link between rs12696304 genotype and chromatin remodelling in EOAD has not been established.

An eQTL signal was also observed for LRRC34 in the cerebellar hemisphere. Although its precise role in telomere biology remains unclear, this nucleolar protein has been associated with genomic stability and the assembly of multiprotein complexes [46,47]. Since the cerebellum is affected during advanced stages of amyloid deposition [48,49], the functional relevance of this signal requires further investigation.

Whether or not rs12696304 influences telomere maintenance or chromatin organisation requires experimental validation.

### 4.6. Integrative Biological Framework

Taken together, these findings support the hypothesis that rs12696304 may act as a regulatory modifier of EOAD susceptibility; however, the underlying biological mechanisms remain hypothetical. Two computationally supported yet experimentally unvalidated possibilities include: (1) modulation of *TERC* expression, potentially affecting telomere maintenance and cellular ageing; and (2) modulation of ACTRT3 expression, potentially influencing chromatin organisation.

Previous studies have shown that *PSEN1* A431E is associated with disrupted calcium homeostasis, mitochondrial dysfunction, and oxidative stress [6,7]. In this context, reduced telomere maintenance capacity could contribute to cellular senescence in glial, immune, and endothelial cells [9,50]. Variants in *TERT* and *TERC* may therefore act as modifiers of biological ageing and disease susceptibility [39]. These proposed mechanisms are inferential, as telomere length was not measured in this study. Accordingly, the integrative framework presented here should be viewed as hypothesis-generating, grounded in genetic association and in silico predictions rather than direct functional evidence. Empirical validation, particularly through direct telomere length quantification and functional studies in EOAD-relevant tissues, will be required to determine whether these putative pathways are biologically operative.

The present findings should also be interpreted within a broader etiological framework. AD and early-onset dementias are complex, multifactorial disorders that result from dynamic interactions between genetic susceptibility and non-genetic factors, including environmental exposures, lifestyle behaviors, and social determinants of health (SDoH). Growing evidence supports a gene–environment interaction model in which factors such as diet, physical activity, psychosocial stress, and cumulative environmental exposures modulate molecular pathways involved in neurodegeneration, including oxidative stress, neuroinflammation, and epigenetic regulation [51,52]. Cardiometabolic conditions and environmental toxicants have been consistently associated with dementia risk, potentially through shared vascular and inflammatory mechanisms [53,54]. In addition, adverse social environments may amplify genetic risk, while favorable lifestyle conditions may partially attenuate it, supporting a cumulative and integrative risk model [55]. Population-level differences in allele frequency and environmental context may also contribute to variability in genetic effect sizes across ethnic groups, underscoring the importance of investigating these interactions in under-represented populations such as the one studied here [56]. The absence of detailed data on dietary patterns and SDoH in the present study is a limitation, as these variables may act as effect modifiers or residual confounders in the observed genetic associations, and their omission may limit the generalizability of the findings.

### 4.7. Population Comparisons

Allele frequency comparisons between the Western Mexico cohort and reference populations from the 1000 Genomes Project (phase 3) reflected expected patterns of human population structure [57]. While some variants differed from those in European, African, or Asian populations, frequencies were generally comparable to those reported for other Latin American groups, consistent with the well-documented admixture patterns in the region [18,19].

### 4.8. Linkage Disequilibrium Analysis

Linkage disequilibrium analysis among TERT variants was performed in the control group. Although some SNP pairs showed statistically significant LD, the low r^2^ values (<0.20) indicate weak correlation between variants, suggesting that they represent largely independent genetic signals [58]. Because r^2^ reflects the predictability between variants, these low values indicate that haplotype analysis would likely provide limited additional information. Therefore, variants were analysed individually rather than as haplotypes.

## 5. Limitations

This study has some important limitations. First, the sample size of the EOAD group was constrained by the low prevalence of the PSEN1 A431E mutation, a typical feature of rare founder-effect diseases. This limited statistical power to detect small effect sizes and increased the risk of type II errors. In addition, the cross-sectional design precludes establishing causal relationships or evaluating the temporal sequence between genetic variation, telomere maintenance, and symptom onset.

Incomplete clinical data also limited multivariable analysis. This reduced model stability and increased the risk of overfitting, while the absence of comparable data in the control group prevented balanced adjustment between cases and controls.

Direct telomere length measurements were not performed in this cohort, which represents a major limitation of the present study, preventing empirical confirmation of the proposed telomeric mechanism for rs12696304.

Finally, the functional evidence derived from bioinformatic resources such as GTEx and RegulomeDB should be interpreted as hypothesis-generating. These databases reflect basal gene expression in the general adult population and may not capture the specific biological context of PSEN1 A431E–associated EOAD.

### Future Perspectives

Future studies should prioritize direct quantification of telomere length—such as by relative qPCR or Southern blot—to determine whether the genotype-level association observed for rs12696304 operates primarily through modulation of TERC expression, ACTRT3, or both. Functional validation in EOAD-relevant tissues, particularly neuronal and glial cell models, will be required to assess whether the eQTL signals identified in peripheral blood and pituitary tissue extend to the central nervous system. Replication of these findings in independent cohorts of PSEN1 A431E carriers, as well as in other familial EOAD populations, is needed to confirm the robustness of the association. Longitudinal study designs integrating genetic, telomeric, epigenomic, and clinical data will be essential to establish the temporal relationship between telomere biology and symptom onset in EOAD. Finally, multi-omics approaches incorporating transcriptomics, chromatin accessibility profiling, and proteomics in EOAD-relevant tissues could help clarify whether rs12696304 acts as a regulatory modifier through chromatin organization, telomere maintenance, or both pathways.

## 6. Conclusions

Most TERT variants did not show significant associations in this cohort, although several were located in genomic regions with consistent regulatory evidence. In contrast, the *rs12696304* (TERC) variant showed a genotype-level association signal and a notable in silico regulatory profile, including eQTL evidence for ACTRT3 and other genes within the TERC locus (3q26.2). The convergence of association patterns, functional annotation, and biological context suggests that this locus warrants further investigation as a potential regulatory modifier in familial EOAD, possibly through mechanisms related to telomere maintenance and epigenomic organization. Although these findings are preliminary and require replication in independent cohorts and direct functional validation, they provide hypothesis-generating evidence that the TERC locus (3q26.2) may be a susceptibility modifier in the context of highly penetrant mutations such as *PSEN1 A431E*.

## Figures and Tables

**Table 1:** Sociodemographic and clinical characteristics of the study participants.

Characteristic	Controls (n = 179)	EOAD Cases (n = 69)
Sex, male, n (%)	90 (50.28)	39 (43.48)
Sex, female, n (%)	89 (49.72)	30 (56.52)
Age at sampling, years, median (IQR)	47 (41–65)	50 (45–51)
Age at symptom onset, years, median (IQR)	NA	42 (39–45)
Disease duration at evaluation, years, mean	NA	7.45

Values are n (%) unless otherwise indicated. Age is reported as median (IQR, 25th–75th percentile). EOAD, early-onset Alzheimer’s disease; NA, not applicable.

**Table 2:** Clinical manifestations among EOAD cases (n = 69).

Domain	Clinical Feature	n (%)
Cognitive and behavioural	Amnesia	23 (33.33)
	Disorientation	17 (24.64)
	Dyscalculia	25 (36.23)
	Aphasia	20 (28.99)
	Mutism	7 (10.14)
	Perseveration	7 (10.14)
	Delirium	10 (14.49)
	Hallucinations	10 (14.49)
	Impaired insight	25 (36.23)
	Emotional lability	29 (42.03)
	Aggressive behaviour	12 (17.39)
Motor (pyramidal)	Paraparesis	12 (17.39)
	Spasticity	16 (23.19)
	Rigidity	16 (23.19)
	Muscle weakness	8 (11.59)
	Gait disturbance	43 (62.32)
	Flaccidity	1 (1.45)
Extrapyramidal/movement	Myoclonus	11 (15.94)
	Tremor	11 (15.94)
	Incoordination	21 (30.43)
Neuropsychiatric	Depression	22 (31.88)
	Anxiety	23 (33.33)
	Apathy	15 (21.74)
	Irritability	42 (60.87)
	Social withdrawal	11 (15.94)
	Panic attacks	3 (4.35)
	Insomnia	35 (50.72)
Other clinical features	Seizures	8 (11.59)
	Urinary incontinence	19 (27.54)
	Weight loss	21 (30.43)

Values are n (%). EOAD, early-onset Alzheimer’s disease.

## Data Availability

The original contributions presented in this study are included in the article/Supplementary Material. Further inquiries can be directed to the corresponding authors.

## Supplementary Materials

The following supporting information can be downloaded at https://www.mdpi.com/article/10.3390/medsci14020228/s1, Figure S1. eQTL analysis for TERT and TERC variants. (A) rs12696304 (TERC) showing dose-dependent effect on the expression of ACTRT3 and LRRC34 in blood, pituitary and cerebellar hemisphere. (B) rs10069690 (TERT) associated with significant changes in TERT expression in caudate and nucleus accumbens. (C) rs2242652 (TERT) showing transcriptional modulation of TERT in the basal ganglia. The p values indicate statistically significant associations between genotype and expression levels. Figure S2. Representation of the binding motif identified in the analyzed sequence. The upper panel shows the consensus nucleotide sequence (5′→3′), highlighting in a black box the evaluated variant position. In the lower panel, the sequence logo indicates the relative frequency and information contribution (in bits) of each nucleotide at each position of the motif. The red box marks the position whose variability presents the greatest potential impact on the affinity and specificity of the regulatory binding site. Figure S3. Regulatory motif and variant position within the predicted binding site. The upper panel shows the consensus sequence (5′→3′) of the identified motif, highlighting with a black box the specific position corresponding to the evaluated variant. In the lower panel, the sequence logo represents the information contribution (in bits) and the frequency of each nucleotide at each position of the motif. The red box indicates the most conserved position within the binding site, suggesting that a change at this base could significantly modify the affinity of the transcription factor for this specific motif. Figure S4. Motif and variant position located at the 3′ end of the predicted binding site. The upper panel shows the consensus sequence (5′→3′) of the identified regulatory motif, with the variable base highlighted by a black box. In the lower panel, the sequence logo represents the information content (in bits) and the relative frequency of each nucleotide at each position of the motif. The red box marks the least conserved position within the motif, located at the 3′ end, suggesting that the variant may exert a weaker regulatory effect compared with highly conserved central positions within the binding site. Table S1. Linkage disequilibrium between analyzed variants (controls).

## Abbreviations

The following abbreviations are used in this manuscript:

AD	Alzheimer’s disease
APC	Article Processing Charge
CI	Confidence interval
DNA	Deoxyribonucleic acid
eQTL	Expression quantitative trait locus
EOAD	Early-onset Alzheimer’s disease
GTEx	Genotype-Tissue Expression
GWAS	Genome-wide association study
HWE	Hardy–Weinberg equilibrium
LD	Linkage disequilibrium
LOAD	Late-onset Alzheimer’s disease
OR	Odds ratio
PCR	Polymerase chain reaction
PSEN1	Presenilin 1
SNP	Single-nucleotide polymorphism
TERC	Telomerase RNA component
TERT	Telomerase reverse transcriptase
TL	Telomere length
