# Supplementary material for: Regulatory Variation at TERT and TERC Shows Limited Association with Early-Onset Alzheimer’s Disease in Carriers of the Mexican Founder Mutation PSEN1 A431E
Source: Med Sci (Basel). 2026 Apr 30;14(2):228. doi: 10.3390/medsci14020228 (PMC13214640; doi:10.3390/medsci14020228)
Supplement: Supplementary file 1 [file medsci-14-00228-s001.zip › medsci-4222458-supplementary.pdf]

medical sciences

Supplementary Materials: Regulatory Variation at TERT and TERC Shows Limited Association with Early-Onset Alzheimer's Disease in Carriers of the Mexican Founder Mutation *PSEN1* A431E

Celeste Patricia Gazcón-Rivas, Iliannis Yisel Roa-Bruzón, Luis Félix Duany-Almira, Cesar Aly Valdéz-Gaxiola, Sofia Dumois-Petersen, Luis Eduardo Figuera-Villanueva, Antonio Quintero-Ramos, Carmen Magdalena Gurrola-Díaz, Daniel Ortuño-Sahagun, Yeminia Valle and Oscar Arias-Carrión

In silico analysis of the variants

The five analyzed variants show relevant regulatory profiles. rs12696304 (TERC) stands out with Rank 1a (315 peaks) and broad enhancer/promoter activity, multiple eQTLs, and strong transcription factor binding, highlighting among them CTCF, a key organizer of chromosomal architecture; POLR2A, the main subunit of RNA polymerase II; GABPB1, CHD4, HDAC2, ZNF217, JUN, TCF12 and TRIM28, all key actors in chromatin remodeling, signaling-dependent transcription, dynamic epigenetic repression and assembly of nuclear regulatory complexes. rs2242652 (Rank 2b) and rs2853677, as well as rs2736100 (Rank 1f), are located in active enhancers of the TERT locus, with evidence of brain accessibility and eQTL function. rs10069690 (Rank 5) shows intermediate epigenomic activity and motif alteration such as KLF10.

eQTL

Figure S1 shows the expression profiles. The graphs show that the SNP rs12696304 (A) acts as an eQTL on ACTRT3 (whole blood and pituitary) and LRRC34 (cerebellar hemisphere). In the three tissues, a consistent pattern of dose-dependent allelic effect (CC > CG > GG) is observed, where the G allele is associated with lower gene expression, with robust significance (p between 10^{-4} and 10^{-5}).

Regarding rs10069690 (B), it acts as an eQTL in the basal ganglia, specifically in caudate and nucleus accumbens. A genotype-dependent pattern (CC, CT, TT) is observed with significant differences in TERT expression ($p \approx 10^{-6}$–10^{-8}).

rs2242652 (C) acts as an eQTL in nucleus accumbens and caudate. The GA genotype shows higher TERT expression compared to GG, while AA —although with low n— maintains the trend, with highly significant associations ($p \approx 10^{-6}$–10^{-5}).

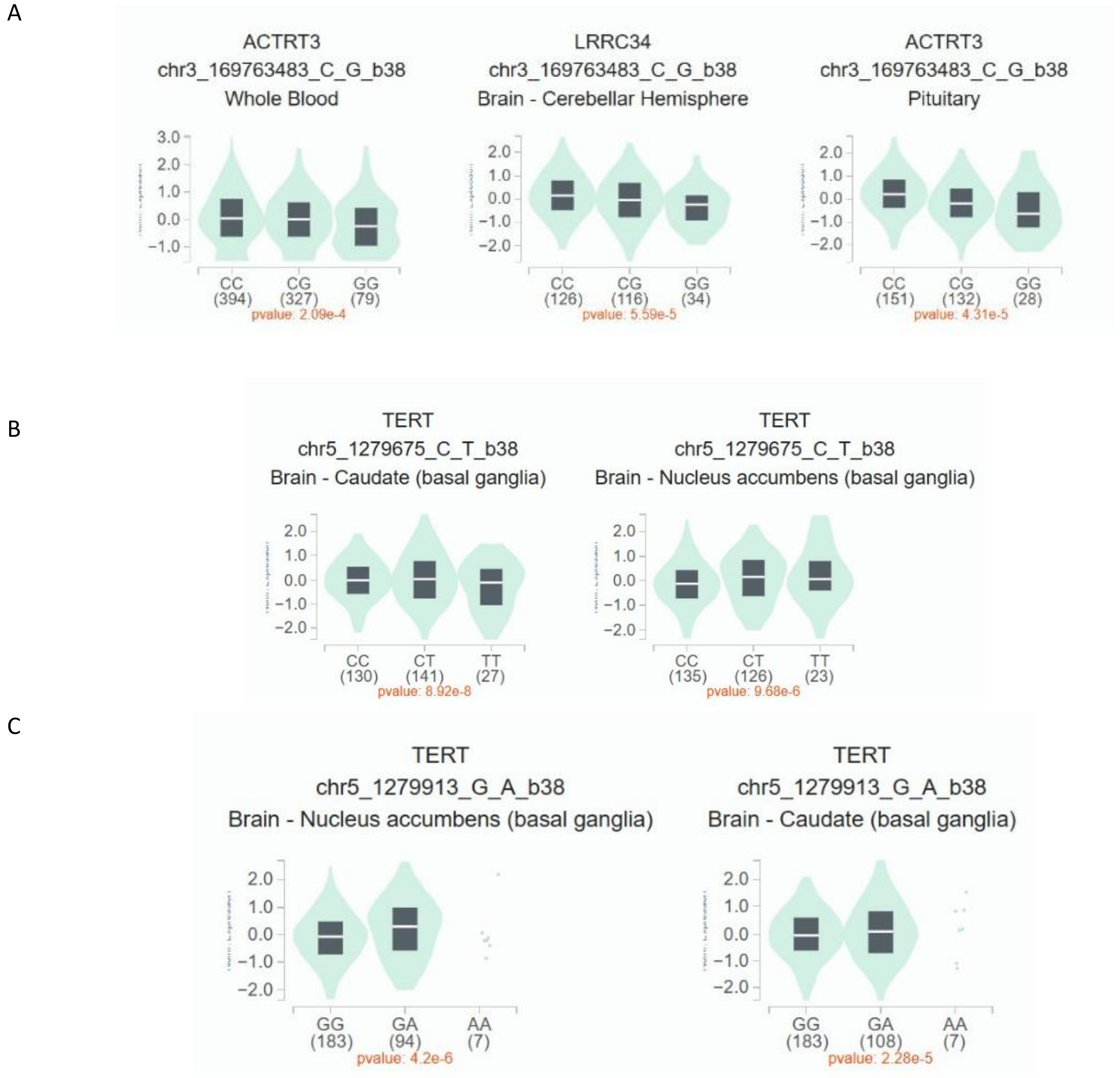

Figure S1. eQTL analysis for TERT and TERC variants. (A) rs12696304 (TERC) showing dose-dependent effect on the expression of ACTRT3 and LRRC34 in blood, pituitary and cerebellar hemisphere. (B) rs10069690 (TERT) associated with significant changes in TERT expression in caudate and nucleus accumbens. (C) rs2242652 (TERT) showing transcriptional modulation of TERT in the basal ganglia. The p values indicate statistically significant associations between genotype and expression levels.

Regulome

The rs2853677 variant, located at chr5:1287078–1287079, presents a RegulomeDB score of 1f, one of the highest categories of functional evidence. This score indicates that the variant is highly likely to be involved in gene regulation, as ranks 1a–1f correspond to variants with strong support for affecting transcription factor binding, altering chromatin accessibility, or modifying gene expression levels (eQTLs). The 1f score specifically indicates evidence that the variant functions as an

eQTL, although no direct transcription factor binding footprint was detected. This situation is common in regions where regulation may depend more on chromosomal structural changes or nucleosomal remodeling rather than a single binding site.

RegulomeDB identifies 42 signals or peaks overlapping this region, suggesting that this position lies within a highly active epigenomic environment. ChIP-seq data detect interaction with the transcription factor SCRT2, a neurogenic factor involved in neuronal differentiation and nervous system developmental dynamics, suggesting a potential regulatory function in neuroectoderm-derived tissues or chromatin remodeling pathways associated with cellular plasticity.

The region displays multiple active chromatin states, including Active Enhancer 2, Genic enhancer 1, Strong transcription, and other transcriptionally active states across several tissues. This indicates that the variant is located within or very close to a functional enhancer, likely one with cell-type–dependent dynamic activity. The multiplicity of chromatin states reflects that the locus is integrated into a complex regulatory architecture, possibly functioning as a gene expression enhancer in multiple biological contexts. The additional presence of Polycomb-repressed states and heterochromatin suggests that the locus may undergo environment-dependent epigenetic changes, alternating between active and inactive states, a pattern typical of genes under tight regulation during development or in specialized tissues.

Regarding chromatin accessibility, RegulomeDB reports 23 DNase-seq/ATAC-seq experiments with positive signal in this region, including frontal cortex (area 46), coronary artery, suprapubic skin, osteoblasts, adipose tissue, pancreatic islets, and several immune cell types (CD4+, CD8+, regulatory T cells). This demonstrates that the locus is not only accessible but also potentially regulatory across multiple human tissues, reinforcing its functional importance. The presence of accessibility in the prefrontal cortex is particularly relevant for this project, given its relationship with neurobiology, neurodegenerative disease, or gene expression modulation in brain tissues.

A key finding is that rs2853677 appears as an eQTL in two tissues: suprapubic skin and lower leg skin, indicating that the allele modulates gene expression in epithelial tissues. Although these tissues are not brain-derived, the presence of eQTL evidence demonstrates that the variant has a real capacity to modify expression levels, consistent with its functional classification of 1f. The absence of reported eQTLs in brain tissues does not invalidate its potential activity there; it simply indicates lack of statistical evidence in GTEx or equivalent datasets. Given that the region is accessible in the frontal cortex, there is biological plausibility that rs2853677 may influence neuronal or glial expression under specific conditions.

Regarding allele frequency, the variant shows a consistent pattern across multiple cohorts (gnomAD, 1000 Genomes, TOPMed, ALSPAC, TWINSUK, Estonia, PAGE), with the A allele being the major allele (≈0.63–0.68) and the G allele being the minor allele (≈0.32–0.44). The variability across populations suggests non-uniform evolutionary pressure, which is typical of variants involved in environment-dependent regulation or local adaptation.

The rs2736100 variant, located at chr5:1286400–1286401, has a RegulomeDB score of 1f, placing it among the noncoding variants with the highest probability of exerting a real regulatory effect on gene expression. The fact that this SNP generates 50 epigenomic peaks across multiple experiments indicates that the region in which it resides is highly active from a regulatory perspective. This position lies within an environment rich in chromatin states associated with enhancers, including Active Enhancer 2, Genic enhancer 1, and regions of Strong transcription, suggesting that rs2736100 is embedded within an enhancer module with functional relevance across multiple cellular contexts.

Unlike other functional SNPs, this variant does not show direct evidence of transcription factor binding (no ChIP-seq hits), but it displays a clear epigenomic pattern of enhancer activity, suggesting that its regulatory impact may depend on chromatin remodeling, long-range chromosomal interactions, or cooperative effects with cofactors that are not typically detected as classical transcription factor footprints.

The variant exhibits high chromatin accessibility, with 30 positive DNase-seq/ATAC-seq signals, including brain tissues such as the posterior cingulate gyrus, middle frontal area 46, and the head of the caudate nucleus, as well as immune cells (B cells), cancer cell lines, osteoblasts, and arterial tissues. The accessibility of this locus in cortical and subcortical regions of the human brain is particularly significant, as it suggests that rs2736100 may participate in transcriptional regulation relevant to neurobiology, synaptic plasticity, or cellular processes associated with brain aging or neurodegenerative disease. The presence of the SNP in regions such as the posterior cingulate and prefrontal cortex (area 46)both regions affected early in cognitive decline—strengthens the biological plausibility of a regulatory role in neuronal pathways.

Regarding population variation, rs2736100 shows highly balanced allele frequencies (C ≈ 0.47, A ≈ 0.53 in gnomAD, 1000G, and TOPMed), a pattern suggestive of evolutionary balance or stabilizing selection in the human population. This genetic equilibrium is often observed in variants that, although not lethal, exert subtle functional effects on gene expression and may influence complex phenotypes such as longevity, cellular proliferation, or polygenic disease risk.

From a functional standpoint, rs2736100 is located within a super-enhancer of the TERT locus, widely documented in the literature as a regulatory hotspot associated with multiple diseases, particularly cancer, cellular proliferative capacity, and telomere length. This SNP has been reported in numerous studies as a critical modulator of TERT expression, acting as an eQTL in several tissues. Although RegulomeDB detects only one eQTL in the current query, independent studies have confirmed its role in tissue-specific regulation. The absence of identified motifs suggests that the functional effect does not depend on the disruption of a single binding site, but more likely on changes in chromatin accessibility, 3D DNA architecture, or the global affinity of the enhancer for the transcriptional machinery.

The rs12696304 variant, located at chr3:169763482–169763483, displays an extremely strong regulatory profile, with a Rank 1a, the highest category in RegulomeDB. This classification indicates direct evidence that the allele alters transcription factor binding and is associated with real changes in gene expression in humans. The region presents 315 epigenomic peaks, an exceptional signal indicating that the SNP lies within a densely occupied regulatory hotspot enriched in transcriptional machinery and cofactors.

From a chromatin perspective, rs12696304 appears in multiple highly active functional states, including Flanking TSS, Active enhancer 1, Active enhancer 2, and Weak enhancer, suggesting that this position forms part of a super-enhancer or extended promoter region involved in fine transcriptional regulation. The strong signal near TSS flanking regions indicates direct proximity to a promoter and therefore potential impact on transcription initiation.

The ChIP-seq profile strongly supports its regulatory function, with 193 transcription factors and regulatory proteins binding at or near the site. Among them are CTCF, a key organizer of chromosomal architecture; POLR2A, the principal subunit of RNA polymerase II; and additional regulators such as GABPB1, CHD4, HDAC2, ZNF217, JUN, TCF12, and TRIM28, all of which play critical roles in chromatin remodeling, signal-dependent transcription, dynamic epigenetic repression, and nuclear regulatory complex assembly. The coincidence of CTCF and POLR2A signals is particularly revealing: CTCF suggests that rs12696304 may modulate topological boundaries (TADs) or enhancer–promoter loops, while POLR2A indicates direct transcriptional activity on a target gene. The presence of chromatin remodelers such as CHD4 and deacetylases such as HDAC2 suggests that this region participates in dynamic activation–repression cycles typical of signal-responsive enhancers.

Although the number of accessibility experiments is relatively low (only two tissues: foreskin fibroblasts and hematopoietic progenitors), this does not reduce the functional relevance of the variant, since chromatin accessibility is highly cell-type specific. Instead, the extremely high density of ChIP-seq data and chromatin states indicates that the locus is highly active across a wide range of transcriptional conditions. Furthermore, the presence of altered motifs for NR1H3 and RXRA

transcription factors, together with four experimental footprints, supports the idea that the allelic change in this SNP directly alters transcription factor binding affinity (Figure 10) and therefore modifies the enhancer or promoter activity of the site.

The set of 19 significant eQTLs associated with rs12696304 is another critical indicator of its real functional impact. These eQTLs appear across a wide diversity of tissues, including gastrocnemius medialis muscle, fibroblasts, ascending aorta, tibial artery, tibial nerve, cerebellum, testis, esophageal muscular tissue, and omental adipose tissue. The broad tissue distribution of expression effects suggests that the variant modulates a gene with systemic roles, likely involved in metabolic, vascular, or neurological regulation. The eQTL signals in cerebellum are particularly relevant in the context of research on neurodegeneration or cellular aging. The global allele frequency (C ≈ 0.61, G ≈ 0.39 in gnomAD and TOPMed) indicates that rs12696304 is a common variant in the human population, allowing small cumulative regulatory effects to potentially contribute to the risk of complex diseases. Figure S2.

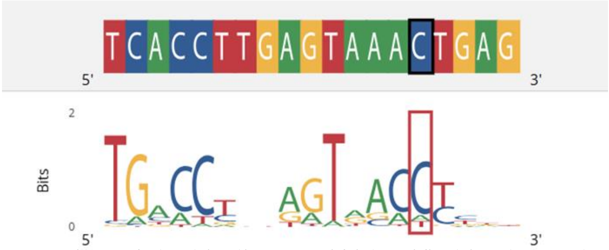

Figure S2. Representation of the binding motif identified in the analyzed sequence. The upper panel shows the consensus nucleotide sequence (5′→3′), highlighting in a black box the evaluated variant position. In the lower panel, the sequence logo indicates the relative frequency and information contribution (in bits) of each nucleotide at each position of the motif. The red box marks the position whose variability presents the greatest potential impact on the affinity and specificity of the regulatory binding site.

The rs10069690 variant, located at chr5:1,279,674, displays an intermediate regulatory profile according to RegulomeDB, with a Rank 5 classification, indicating moderate evidence that this SNP may influence regulatory elements, although with less direct functional support compared with variants classified in categories 1–3. Despite this, the site is characterized by a high epigenomic density with 121 detected peaks, revealing a complex regulatory environment where multiple chromatin states converge, including Active Enhancer 2, Genic Enhancer 1, Strong transcription, and Weak transcription. This diversity suggests that rs10069690 resides in a dynamic genomic segment involved in both transcriptional enhancement and intragenic regulation of nearby genes, particularly those within the TERT locus, which has been widely associated with longevity, cancer, and telomere maintenance.

Although no direct positive ChIP-seq experiments were detected, the SNP lies within regions of open chromatin accessibility in 61 tissues and cell lines, including spleen, left and right heart ventricles, frontal cortex area 46, placenta, aorta, lung, and pancreas. This indicates that the allele affects an active regulatory element across multiple cellular types, particularly in cardiovascular, pulmonary, and immune tissues.

The presence of an altered functional motif for KLF10, accompanied by 24 footprints, is particularly noteworthy (Figure S3), because KLF10 is a key transcription factor involved in stress responses, TGF-β signaling regulation, and tissue homeostasis. The existence of experimental footprints implies that rs10069690 physically modifies the binding affinity of KLF10 in human primary cells, which could alter the expression of specific genes depending on the allele present.

Although this SNP does not show eQTL associations within the datasets included in RegulomeDB, its active epigenomic context—together with previous studies linking rs10069690 with susceptibility to breast cancer, renal cancer, and telomere length—supports the notion that this variant may exert an indirect regulatory effect, possibly dependent on cellular context or specific environmental signals.

With balanced allele frequencies (C ≈ 0.65; T ≈ 0.35), rs10069690 represents a common variant that may act as a subtle but relevant modulator of gene regulation at the TERT locus, particularly under conditions involving cell proliferation, inflammation, or tissue remodeling. Overall, rs10069690 does not exhibit the strong direct regulatory evidence observed in Rank 1 variants; however, its location within an active enhancer environment, its high accessibility across multiple tissues, and the demonstrated alteration of a KLF10 binding site indicate that it likely has a real and biologically plausible functional impact, with potential relevance in aging processes, cancer biology, and cellular homeostasis.

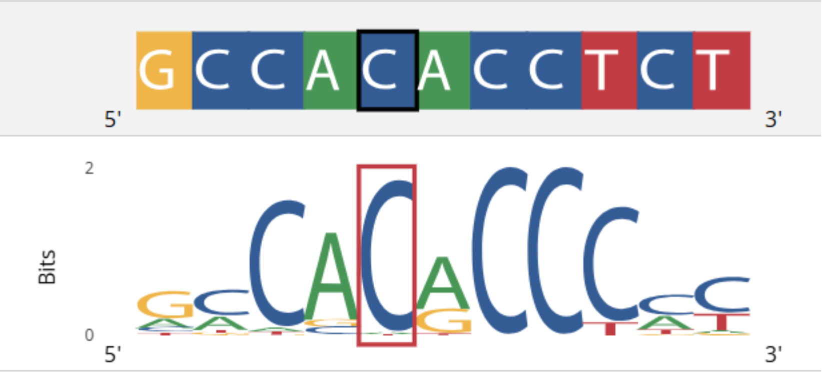

Figure S3. Regulatory motif and variant position within the predicted binding site. The upper panel shows the consensus sequence (5′→3′) of the identified motif, highlighting with a black box the specific position corresponding to the evaluated variant. In the lower panel, the sequence logo represents the information contribution (in bits) and the frequency of each nucleotide at each position of the motif. The red box indicates the most conserved position within the binding site, suggesting that a change at this base could significantly modify the affinity of the transcription factor for this specific motif.

The rs2242652 variant, located at chr5:1,279,912, presents a RegulomeDB Rank 2b classification, indicating strong functional evidence that this SNP affects gene regulation through alterations in transcription factor binding sites with direct experimental support (ChIP-seq + altered motif). This score suggests a greater potential biological impact than variants classified in categories 4–6, since a Rank 2 classification implies that the variant overlaps with a transcription factor motif and that altered experimental binding has been detected for at least one regulatory protein.

The site lies within a highly active genomic region, with 100 regulatory peaks, reflecting a dynamic enhancer environment and active transcription characteristic of the TERT locus. Chromatin in this region exhibits diverse epigenomic states, including Active Enhancer 2, Genic Enhancer 1, Strong transcription, and Weak transcription, indicating that rs2242652 resides within a regulatory landscape crucial for the fine control of TERT expression, particularly in proliferative cells.

The variant displays open chromatin accessibility across 55 human tissues and cell types, including posterior cingulate cortex, spleen, right heart ventricle, caudate nucleus, lung, placenta, descending colon, aorta, frontal cortex, and ovary, demonstrating that the site is functional in multiple biological contexts, including neurobiology, inflammation, immunity, cardiac metabolism, and reproductive biology.

Importantly, RegulomeDB identifies experimental binding for NFIB and NFIC, transcription factors that play critical roles in cell differentiation, proliferation, and cellular remodeling. These proteins belong to the Nuclear Factor I family, known to regulate enhancers, chromatin architecture, and genes associated with cancer and tissue regeneration.

Additionally, the detection of footprints and one motif for ZFX (Figure S4)—a transcription factor essential for stem cell maintenance and self-renewal—together with 11 experimental footprints, suggests that rs2242652 may directly alter the binding affinity of ZFX, thereby modifying the regulation of TERT in progenitor cells and highly proliferative cell lines.

Furthermore, allele frequency data show a major allele (G ≈ 0.83) and a minor allele (A ≈ 0.17), indicating that rs2242652 is a common variant acting as a subtle modulator of chromatin regulation at the TERT locus rather than a rare disruptive mutation, but with potential cumulative effects on longevity, cellular proliferation, and cancer risk.

Although specific eQTL associations were not observed in the evaluated databases, the combination of open chromatin accessibility, active enhancer states, altered binding of NFIB/NFIC, and functional ZFX footprints indicates that rs2242652 represents a genuine regulatory variant, likely involved in fine-tuning TERT expression in a tissue-, proliferation-, and signaling-dependent manner.

Overall, this profile suggests that rs2242652 functions as a relevant regulatory node in the transcriptional control of the TERT gene, with potential implications in aging, telomere biology disorders (telomeropathies), and cancer susceptibility.

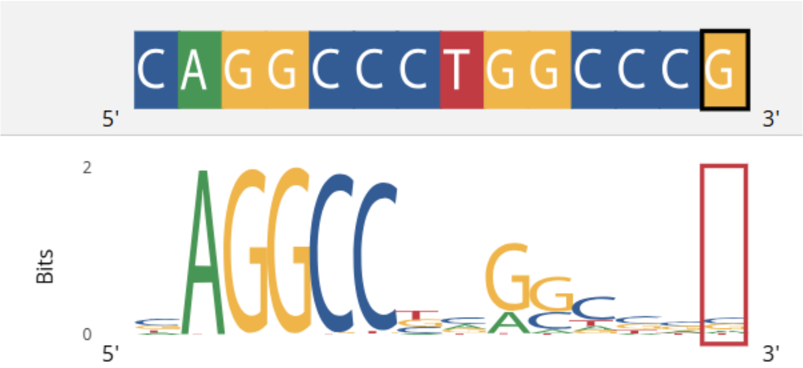

Figure S4. Motif and variant position located at the 3′ end of the predicted binding site. The upper panel shows the consensus sequence (5′→3′) of the identified regulatory motif, with the variable base highlighted by a black box. In the lower panel, the sequence logo represents the information content (in bits) and the relative frequency of each nucleotide at each position of the motif. The red box marks the least conserved position within the motif, located at the 3′ end, suggesting that the variant may exert a weaker regulatory effect compared with highly conserved central positions within the binding site.

Linkage disequilibrium among TERT variants was evaluated exclusively in controls using D′ and r^2 statistics. (Lewontin 1964) Variants demonstrating consistently low r^2 values (<0.20) were interpreted as weakly correlated, supporting independent variant-level analyses (Table S1). Haplotype modelling was not performed due to the limited predictive correlation between loci.

Table S1. Linkage disequilibrium between analyzed variants (controls).

SNP 1	SNP 2	D′	r^2	P value (LD, df = 1)	Interpretation
**rs2242652**	rs2853677	0.253	0.0225	0.0435	Low
**rs2242652**	rs10069690	0.520	0.1539	1.3×10^{-7}	Low–moderate
**rs2242652**	rs2736100	0.521	0.0652	5.9×10^{-4}	Low
**rs2853677**	rs10069690	0.195	0.0235	0.0392	Low
**rs2853677**	rs2736100	0.444	0.1340	8.4×10^{-7}	Low
**rs10069690**	rs2736100	0.428	0.0774	1.8×10^{-4}	Low

D′ and r^2 values are shown together with χ^2 P values for LD (1 degree of freedom). Low r^2 values (< 0.20) indicate limited correlation between variants, even when D′ is moderate. LD categories were assigned based on r^2.
